# Temporal Changes in Serum S100B Levels From Prehospital to Early In-Hospital Sampling in Patients Suffering Traumatic Brain Injury

**DOI:** 10.3389/fneur.2022.800015

**Published:** 2022-04-08

**Authors:** Sophie-Charlott Seidenfaden, Julie Linding Kjerulff, Niels Juul, Hans Kirkegaard, Mette Fogh Møller, Anna-Marie Bloch Münster, Morten Thingemann Bøtker

**Affiliations:** ^1^Research and Development, Prehospital Emergency Medical Services, Aarhus, Denmark; ^2^Neurointensive Care Unit, Department of Anaesthesiology and Intensive Care, Section North, Aarhus University Hospital, Aarhus, Denmark; ^3^Research Centre for Emergency Medicine, Aarhus University Hospital, Aarhus, Denmark; ^4^Department of Clinical Medicine, Aarhus University, Aarhus, Denmark; ^5^Department of Clinical Biochemistry, Regional Hospital West Jutland, Herning, Denmark; ^6^Department of Clinical Biochemistry, Hospital of South West Jutland, Esbjerg, Denmark

**Keywords:** S100B, traumatic brain injury, intracranial lesion, temporal changes, consecutive sampling

## Abstract

**Background:**

The biomarker S100B is used for the rule-out of intracranial lesions in patients with mild traumatic brain injury (TBI) and is suggested for prehospital use in Europe. Early kinetics of S100B are not exhaustively investigated in human TBI. This *post hoc* descriptive study of the data from the PreTBI studies aimed to characterize the early temporal changes of S100B using two-sample timepoints.

**Materials and Methods:**

Two consecutive blood samples were taken prehospital and in-hospital after injury and assayed for S100B. The endpoint adjudication of the outcome intracranial lesion was done by the evaluation of electronic medical patient journals. The data were analyzed using descriptive statistics, scatterplots, and temporal changes estimated by the locally weighted scatterplot smoothing (LOWESS) regression line.

**Results:**

A total of 592 adult patients with TBI were included; 566 with Glasgow Coma Scale (GCS) 14-15, 20 with GCS 9-13, and 6 with GCS 3-8. Intracranial lesions were diagnosed in 44/566 (7.4%) of patients. In 90% of patients, S100B concentrations decreased from prehospital to in-hospital sampling. The mean decrease was−0.34 μg/L. S100B concentrations seem to decline already within 60 min. Patients sampled very close to trauma and patients suffering intracranial lesions may express a slight incline before this decline. Temporal changes of S100B did not differ in patients >65 years of age, in antiplatelet/-coagulant treatment, alcohol intoxicated, or suffering extra-cranial injuries.

**Conclusion:**

S100B concentrations may peak earlier than expected from previous studies of temporal changes in human TBI. Patterns of S100B stand robust to parameters stated as limiting factors to the use for early rule-out of intracranial lesions in the current guidelines. Further studies are needed to investigate the ultra-early temporal profiles of other novel TBI biomarkers to assess prehospital applicability and optimal diagnostic performance in TBI.

## Introduction

Head trauma can cause a spectrum of injuries from simple concussions to severe traumatic brain injury (TBI) (1). TBI is a major cause of death and acquired disability across all ages worldwide. It is a time-critical condition and prehospital triage of high-risk patients to specialized neurosurgical treatment is essential (2, 3). The Glasgow Coma Scale (GCS) score acts as a predictor of intracranial lesions and mortality following TBI (4–7). However, TBI causes diverse symptoms in the initial acute post-trauma phase. Clinical assessment can be indecisive and supportive tools for prehospital risk stratification and triage are needed.

The biomarker S100 calcium-binding protein B (S100B) is a protein located in the glial cells of the central and peripheral nervous system (8). It was implemented in the 2014 Scandinavian Neurotrauma Committee guideline for early in-hospital rule-out of the intracranial lesion in adult patients with mild TBI (9, 10). S100B concentrations are detectable in the peripheral circulation as early as 15 min after trauma with a half-life reported in the range of 1–6 h (8, 11, 12). No studies have examined the temporal changes and trajectories of S100B in very early sampling done <1 h of trauma in patients suffering TBI (13).

By the *post hoc* analysis of data from the PreTBI studies, the overall aim of this study was to characterize the early temporal changes in S100B levels. The specific aims were:

- To describe differences in baseline demographics and clinical characteristics and outcome (being intracranial lesions, need for neurosurgical treatment, and/or death within 7 days of trauma) of patients with mild, moderate, and severe TBI stratified by initial prehospital GCS.
- To describe patterns in S100B concentrations of ultra-early sampling at two consecutive time points of patients with TBI experiencing guideline limitations to the use of S100B for rule-out of intracranial lesions (age >65 years, antiplatelet/-coagulant treatment, alcohol consumption prior to trauma, tubular bone fracture).
- To describe the temporal changes and trajectory of S100B development in ultra-early stages after head trauma in relation to initial GCS and the clinical outcome being (1) intracranial lesions, (2) need for neurosurgical observation and/or neurosurgical intervention, and (3) death within 7 days of trauma due to TBI.

## Materials and Methods

### Study Design

In this current descriptive study, we present results from a *post hoc* analysis of the three PreTBI studies I, II, and III (ClinicalTrials.gov identifier: NCT03028376, NCT02867137, and NCT03062566) which were GCS stratified studies in patients with head trauma conducted as investigator-driven, outcome assessor-blinded, prospective, observational, multi-center, cohort studies in the Central Denmark Region from 15 February 2017 to 01 February 2019.

PreTBI I (enrolling mild TBI patients, initial GCS 14-15) was the main study, and diagnostic accuracy data has previously been published (13). Inclusion was performed as consecutive sampling and ended at sample size saturation. PreTBI II (enrolling moderate TBI patients, initial GCS 9-13) and PreTBI III (enrolling severe TBI patients, initial GCS 3-8) were conducted as convenience samples in relation to the PreTBI I study. The sample size was determined by power calculation in relation to the PreTBI I study (13).

### Participants and Setting

Eligible patients were adults ≥ 18 years of age suffering head trauma receiving ambulance dispatch from the Prehospital Emergency Medical Services, Central Denmark Region. Inclusion criteria were relevant head trauma (blunt or penetrating) and GCS 14-15 with signs of commotio cerebri (loss of consciousness for <30 min *and/or* alteration of mental state: being dazed, confused or disorientated, *and/or* loss of memory for events immediately before/after trauma) or GCS 9-13 or GCS 3-8. The exclusion criteria were an unknown time of trauma, >6 h elapsed from trauma, invalid blood sampling, incomplete registration to the database, foreign citizen, known dementia, chronic psychosis, and/or active central nervous system (CNS) pathology.

The Central Denmark Region is inhabited by 1.3 million people (23% of the total Danish population) and is one of five administrative regions in Denmark. The Danish National Health Service is tax-financed, all prehospital emergency medical services are subsidized, and dispatch is coordinated through one regional Emergency Medical Coordination Center. Within the Central Denmark Region, there are four regional hospitals (West Jutland, Randers, Viborg, and Horsens) with emergency departments and one university hospital (Aarhus) with a major trauma center and neurosurgical facilities. All five centers participated in the PreTBI studies.

Patients were included and consented to the scene of the accident by ambulance personnel. Patents able to consent (suffering mild TBI) gave oral and written informed consent prior to inclusion. For patients unable to consent (suffering moderate and severe TBI), oral and written informed consent was obtained from the participants or next of kin on the following weekday after inclusion pursuant to Danish legislation.

Following on-scene inclusion, prehospital blood sampling was performed. Patients were subsequently transferred to one of the five emergency hospitals, where in-hospital blood sampling was performed.

### Biomarkers and Biochemical Analysis

The prehospital blood sample was collected from a routinely inserted peripheral venous catheter into a 5.5 ml Sarstedt Monovette^®^ (Sarstedt AG & Co., Nümbrecht, Germany) for serum within 6 h of trauma. It was transported by ambulance and picked up by a laboratory technician, who also performed in-hospital sampling. The in-hospital sample was collected into a 6 ml Beckton Dickinson^®^ (Beckton, Dickinson and Company, New Jersey, USA) serum tube and handled under standardized preanalytical conditions.

Samples were centrifuged at 2,200 g for 10 min within 4 h. Until centrifugation, the prehospital sample was kept at ambient temperature in the ambulance until arrival at the hospital, whereas the in-hospital sample was kept at room temperature the entire time. Serum was aliquoted into 4 CryoPure (Sarstedt AG & Co., Nümbrecht, Germany) 1 ml vials containing 0.5 mL each and stored at −80°C.

Biochemical analysis was performed at the Department of Clinical Biochemistry, Regional Hospital West Jutland, Herning. Samples were analyzed within 6 months of collection in blinded batches. Serum S100B concentrations were measured in singular with a routine automated Cobas S100 chemiluminescence immunoassay using a Cobas^®^ e602 analyzer (Roche Diagnostics, Mannheim, Germany). The lower limit of detection was 0.005 μg/l and the analytical imprecision was 1.6–7.1% (monthly mean coefficient of variance for internal routine controls).

Interference indices (Hemolysis, Icterus, and Lipemia) were measured in all samples using the saline protocol on an Alinity^®^ c analyzer (Abbott Diagnostics, Illinois, USA) after two freeze-thaw cycles. Only Hemolysis Index results are reported. According to the manufacturer, S100B measurements are unaffected by hemolysis ] <10 g/l (14).

### Variables and Outcome Measures

In this *post hoc* study, the primary outcome was the association between time from trauma to sampling and serum S100B concentrations.

An outcome assessor committee of two senior researchers (MTB, NJ) blinded to S100B concentrations evaluated all patient courses from medical records and the Cause of Death Register. The assessors evaluated if the patients experienced any of the three outcomes: (1) intracranial lesions identified on cerebral CT examination (subdural, epidural, subarachnoid, and intracerebral hemorrhage, cerebral edema, pneumocephalus, cerebral contusion, and/or skull cap/base fractures), (2) need for neurosurgical observation (admission to neurointensive care unit without prior neurosurgical intervention) and/or neurosurgical intervention (craniotomy, elevation of skull fracture and/or intracranial pressure monitoring) within 7 days of trauma, and (3) death within 7 days of trauma due to TBI. In cases of discrepancy in outcome assessment, a consensus was reached by discussion. In the case that consensus could not be reached, a third assessor was involved and the majority vote was accepted as final.

S100B concentrations are reported and plotted as (1) concentrations, (2) means, (3) dichotomized above/below the predefined cut-off [0.10 μg/L, guideline standard (9)], and (4) S100B (difference in μg/L between prehospital and inhospital concentrations).

### Data Sources

Ambulance personnel registered initial prehospital data (GCS, time of trauma, trauma mechanism, suspected alcohol intake, known antiplatelet/-coagulant treatment, and timepoint of prehospital sampling) in the PreTBI Database provided by TrialPartner^®^ (Lucidity, Dept. of Clinical Medicine, Aarhus University, Denmark). Specifically, the time of trauma was registered as informed by the patient or bystanders on the scene. Missing time stamps from ambulance personnel registrations were supplemented from prehospital and/or in-hospital electronic medical records if possible and otherwise reported as missing. Medical history, prescription medication, trauma mechanism, alcohol consumption, cerebral CT examinations, neurosurgical interventions, intensive care unit admission, and the final diagnosis was extracted from the electronic in-hospital medical records.

Demographic data, vital status, comorbidities for Charlson Comorbidity Index score calculation, and cause of death were extracted from the Danish National Patient Registry, The Danish Causes of Death Register, and The Central Person Registry administered by The Danish Health Data Authority (15–17). All Danish citizens have a unique social security number (CPR number) making it possible to link Danish registers on an individual level.

### Statistical Methods

Data were analyzed using descriptive statistics and were assessed for distribution and variance. Categorical data are presented as numbers and proportions. Continuous data are presented as means with 95% CI or medians and interquartile ranges (IQR) according to distribution. Normality was assessed by visual inspection of histograms and QQ-plots. S100B concentrations were log-transformed to meet linearity prior to analysis. Individual graphics display S100B serum concentrations over time, both as trends of prehospital vs. in-hospital samples and as all concentrations and timepoints overlaid with the locally weighted scatterplot smoothing (LOWESS) regression line. All statistical analysis was done using STATA© intercooled, version 15, (StataCorp LP, College Station, Texas, USA). Missing data were not imputed.

### Ethics

Approvals were obtained from the Danish Data Protection Agency (approval no. 1-10-72-379-16) and the Regional Scientific Ethical Committee System (approval no. 1-10-72-107-16, 1-10-72-108-16, and 1-10-72-109-16) prior to the initiation of the study. The study was performed in accordance with the Declaration of Helsinki. Participants were not exposed to additional invasive procedures or alteration of treatment.

Participation in the study did not cause patient- or system-delay. All patients received usual care regardless of study participation. Treating physicians were not aware of S100B values and study participation did not affect the decision on diagnostic CT examinations. The participants did not experience any physical or physiological advantage or disadvantage by participating. The participants can, at any time, withdraw from the study. Participants did not receive any compensation or fee for participation in the project.

## Results

### Characteristics and Clinical Outcome of Study Subjects

**Figure 1:**
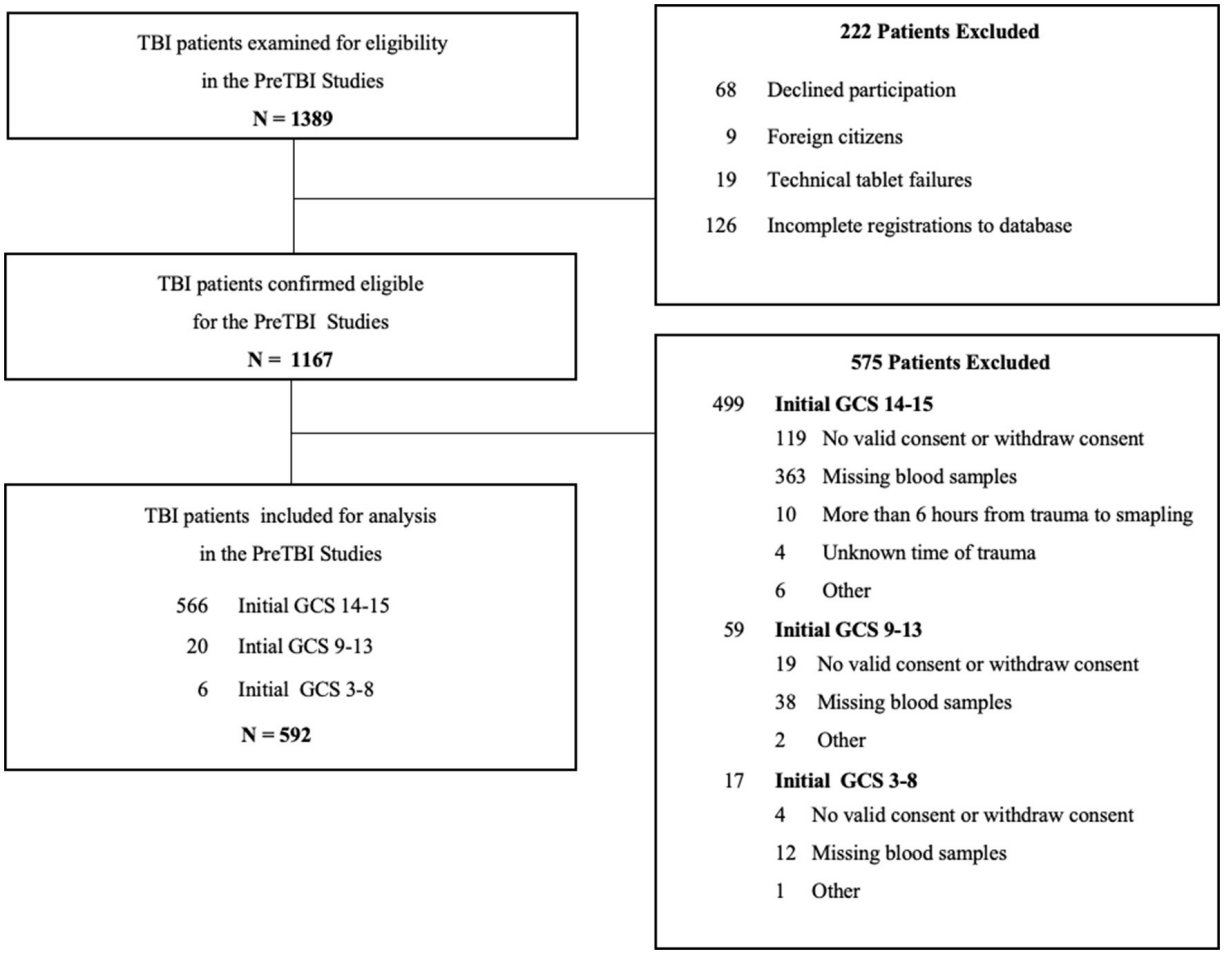
Flow diagram of patient inclusion and exclusion in the PreTBI studies.

Figure 1 displays the inclusion and exclusion of patients. A total of 1,389 patients with TBI were assessed for eligibility and 592 (50.7%) were included. Of these, 566 (95.6%) patients initially presented with GCS 14-15, 20 (3.4%) with GCS 9-13 and 6 (1.0%) with GCS 3-8.

Overall baseline demographics, clinical characteristics, and outcomes are presented in Table 1.

**Table 1:** Demographics, clinical characteristics, and outcome data of the included patients with traumatic brain injury (TBI) stratified by the Glasgow Coma Scale (GCS), *N* = 592.

**Variables**	**Initial GCS 14-15 (mild) *N =* 566**	**Initial GCS 9-13 (moderate) *N* = 20**	**Initial GCS 3-8 (severe) *N =* 6**
**Demographics**			
Age, years, median [IQR]	62 [45;74]	69 [38;76]	41 [35;54]
– > 65 years, n/N (%)	257/566 (45.4)	12/20 (60.0)	1/6 (16.7)
Male, n/N (%)	329/566 (58.1)	13/20 (65.0)	5/6 (83.3)
**Clinical characteristics**			
*Charlson comorbidity index*			
0, n/N (%)	161/566 (28.4)	6/19 (31.6)	3/6 (50)
1–2, n/N (%)	138/566 (24.4)	6/19 (31.6)	2/6 (33.3)
3–4, n/N (%)	159/566 (28.1)	5/19 (26.3)	1/6 (7, 16)
≥5, n/N (%)	108/566 (19.1)	2/19 (10.5)	0/6 (0.0)
*Antiplatelet/-coagulant treatment*			
Yes, n/N (%)	144/556 (25.9)	4/19 (21.0)	0/6 (0.0)
– Acetylsalicylic acid, n/N (%)	65/144 (45.1)	2/4 (50.0)	-
– ADP-receptor antagonist, n/N (%)	32/144 (22.2)	0/4 (0.0)	-
– Vitamin K antagonist, n/N (%)	18/144 (12.5)	2/4 (50.0)	-
– NOAC, n/N (%)	32/144 (22.2)	0/4 (0.0)	-
*Trauma mechanism*			
Traffic, n/N (%)	143/557 (25.6)	4/19 (21.0)	3/6 (50.0)
Fall <2m, n/N (%)	350/557 (62.8)	9/19 (47.4)	2/6 (33.3)
Fall >2m, n/N (%)	17/557 (3.1)	3/19 (15.8)	1/6 (16.7)
Violence, n/N (%)	14/557 (2.5)	0/19 (0.0)	0/6 (0.0)
Other, n/N (%)	33/557(5.9)	3/19 (15.8)	0/6 (0.0)
*Additional symptoms*			
Loss of consciousness and/or amnesia, n/N (%)	142/566 (25.1)	-	-
Vomiting, n/N (%)	23/566 (4.1)	-	-
*Suspected intake of alcohol/drugs prior to trauma*			
Yes, n/N (%)	161/521 (30.9)	6/18(33.3)	3/4 (75.0)
*Bone fracture*			
Yes, n/N (%)	21/566 ()	0/20 (0)	0/6 (0)
*CT examination*			
Yes, n/N (%)	344/566 (60.7)	17/20 (85.0)	5/6 (83.3)
**Clinical outcome**			
Intracranial lesion	32/566 (5.7)	8/20 (40.0)	4/6 (66.7)
– Neurosurgical observation and/or intervention	4/32 (12.5)	5/8 (62.5)	4/4 (100.0)
– Death within 7 days due to TBI	2/566 (0.4)	1/20 (5.0)	0/6 (0.0)

*TBI, Traumatic Brain Injury; GCS, Glasgow Coma Scale; IQR, Interquartile range*.

A total of three patients died due to TBI within 7 days of trauma; two initially presented with GCS 14-15 (Patient 1: multiple brain bleeds, prehospital S100B 0.519 μg/l, and in-hospital 0.149 μg/l, Patient 2: subarachnoid hemorrhage, prehospital S100B 1.31 μg/l and in-hospital 0.373 μg/l respectively) and one with GCS 9-13 (Patient 3: subarachnoid hemorrhage, prehospital S100B 9.62 μg/l and in-hospital 8.05 μg/l).

### S100B Concentrations

**Figure 2:**
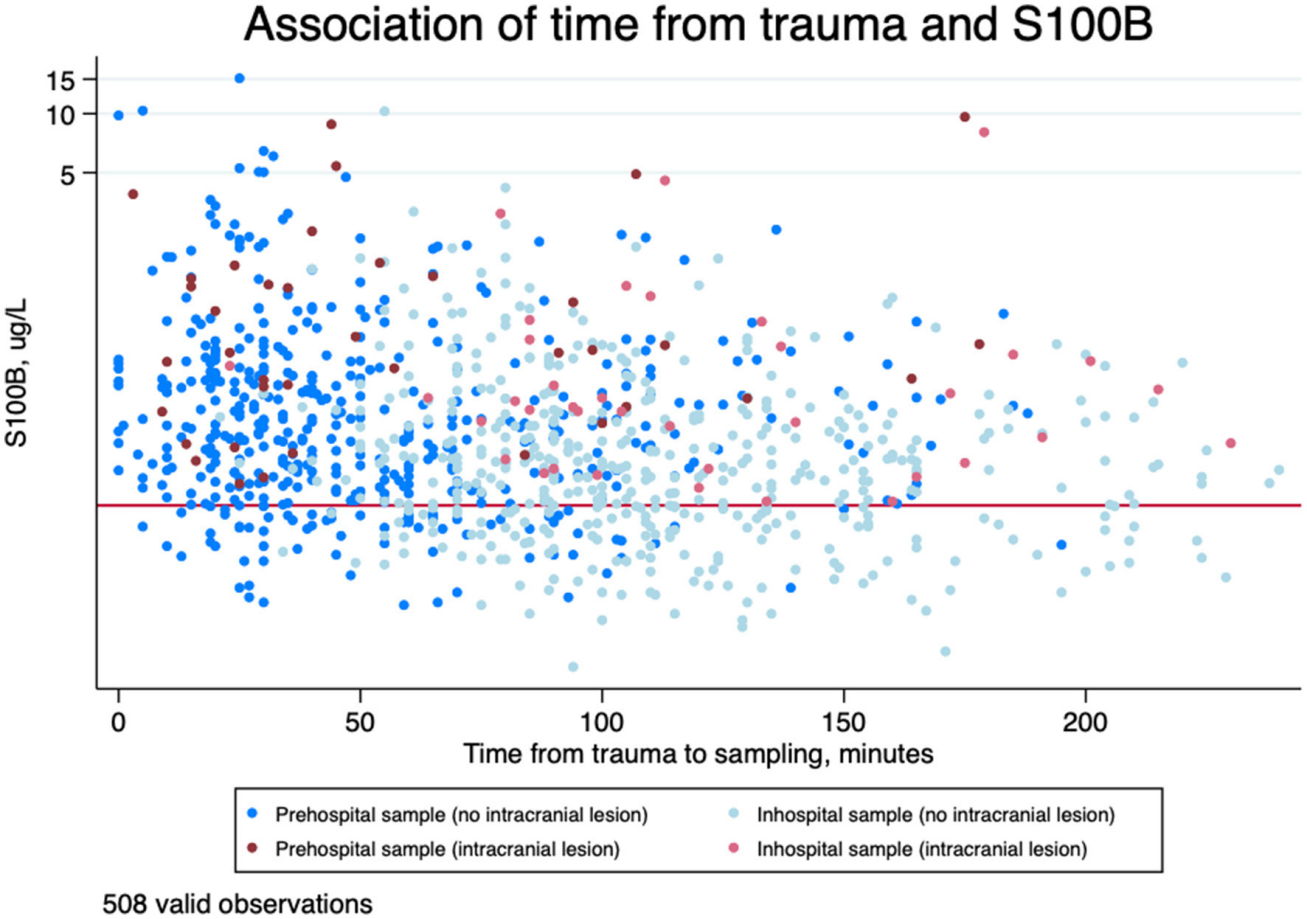
Scatterplot of time from trauma to blood sampling in minutes on an ordinary scale (the first 4 h) and serum S100B concentrations in μg/L on a logarithmic scale in patients with and without intracranial lesion. The red line is indicating guideline cut-off at 0.10 μg/l. *N* = 508 (valid timestamps within the first 4 h).

**Figure 3:**
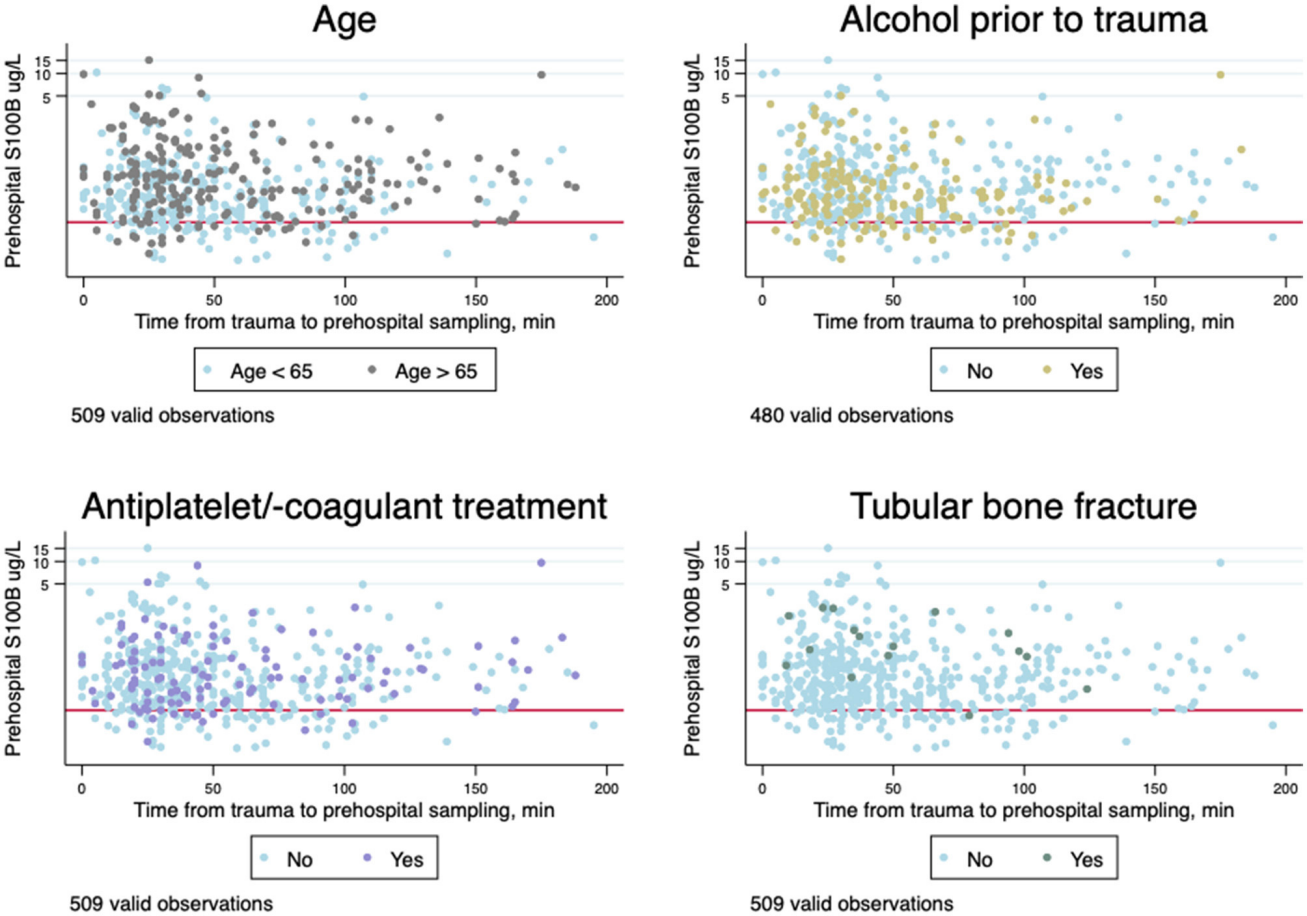
Scatterplots of time from trauma to blood sampling in minutes (the first 4 h) on ordinary scale and prehospital serum S100B concentrations in μg/l a logarithmic scale stratified by age above/below 65 years, consumption of alcohol prior to trauma, antiplatelet/-coagulant treatment, and tubular bone fracture in addition to traumatic pbrqain injury (TBI). The red lines indicate guideline cut-off at 0.10 μg/l.

The time from trauma to sampling, mean S100B concentrations, and dichotomized S100B values above/below 0.10 μg/l stratified by initial GCS are presented in Table 2. All samples contained values above the limit of detection. Prehospital mean S100B concentrations were higher than in-hospital mean S100B concentrations in all GCS groups. The association between prehospital and in-hospital sampling times and S100B concentrations in patients with and without intracranial lesions is displayed in Figure 2 and stratified by GCS in Supplementary Figure S1 (Supplementary Material 1). All patients suffering from intracranial lesions presented with prehospital S100B values above the guideline cut-off > 0.10 μg/l. Temporal changes of prehospital S100B concentrations stratified by factors that may affect rule-out of intracranial lesions using S100B (age > 65 years and antiplatelet/-coagulant treatment, alcohol consumption prior to trauma, and tubular bone fracture in addition to TBI) is displayed in Figure 3. In patients suffering tubular bone fractures in addition to TBI (*n* = 21, all GCS 14-15), mean prehospital and in-hospital S100B concentrations were 0.73 μg/l (95%CI 0.47; 1.14) and 0.39 μg/l (95%CI 0.26; 0.58) respectively.

**Table 2:** S100B concentrations and sampling times in prehospital and in-hospital serum samples from patients with TBI stratified by GCS, *N* = 592.

	**Prehospital**			**Inhospital**		
**Variable**	**Initial GCS 14-15 (mild)**	**Initial GCS 9-13 (moderate)**	**Initial GCS 3-8 (severe)**	**Initial GCS 14-15 (mild)**	**Initial GCS 9-13 (moderate)**	**Initial GCS 3-8 (severe)**
*Valid biomarker concentrations*	*N =* 566	*N =* 20	*N =* 6	*N =* 566	*N =* 20	*N =* 6
S100B μg/L, mean (95%CI)	0.29 (0.26;0.32)	0.51 (0.24;1.1)	0.81 (0.28;2.3)	0.17 (0.16;0.18)	0.33(0.16;0.70)	0.44 (0.17;1.17)
S100B ≥0.10 μg/L, n/N (%)	484/566 (85.5)	18/20 (90.0)	6/6 (100.0)	398/566 (70.3)	16/20 (80.0)	6/6 (100.0)
Hemolysis Index, mean (95%CI)	0.21 (0.20;0.23)	0.23 (0.12;0.43)	0.42 (0.11;1.6)	0.05 (0.05;0.06)	0.08 (0.04;0.2)	0.06 (0.06;0.19)
*Valid sampling times*	*N =* 507	*N =* 16	*N =* 5	*N =* 507	*N =* 16	*N =* 5
Minutes from trauma to sampling, median [IQR]	40 [25;75]	47.5 [32;97]	32 [10;49]	106 [82;146]	106 [80;142]	95 [94;103]

*TBI, Traumatic Brain Injury; GCS, Glasgow Coma Scale; CI, Confidence interval; IQR, Interquartile range*.

### Trajectory and Temporal Changes in S100B Levels

**Figure 4:**
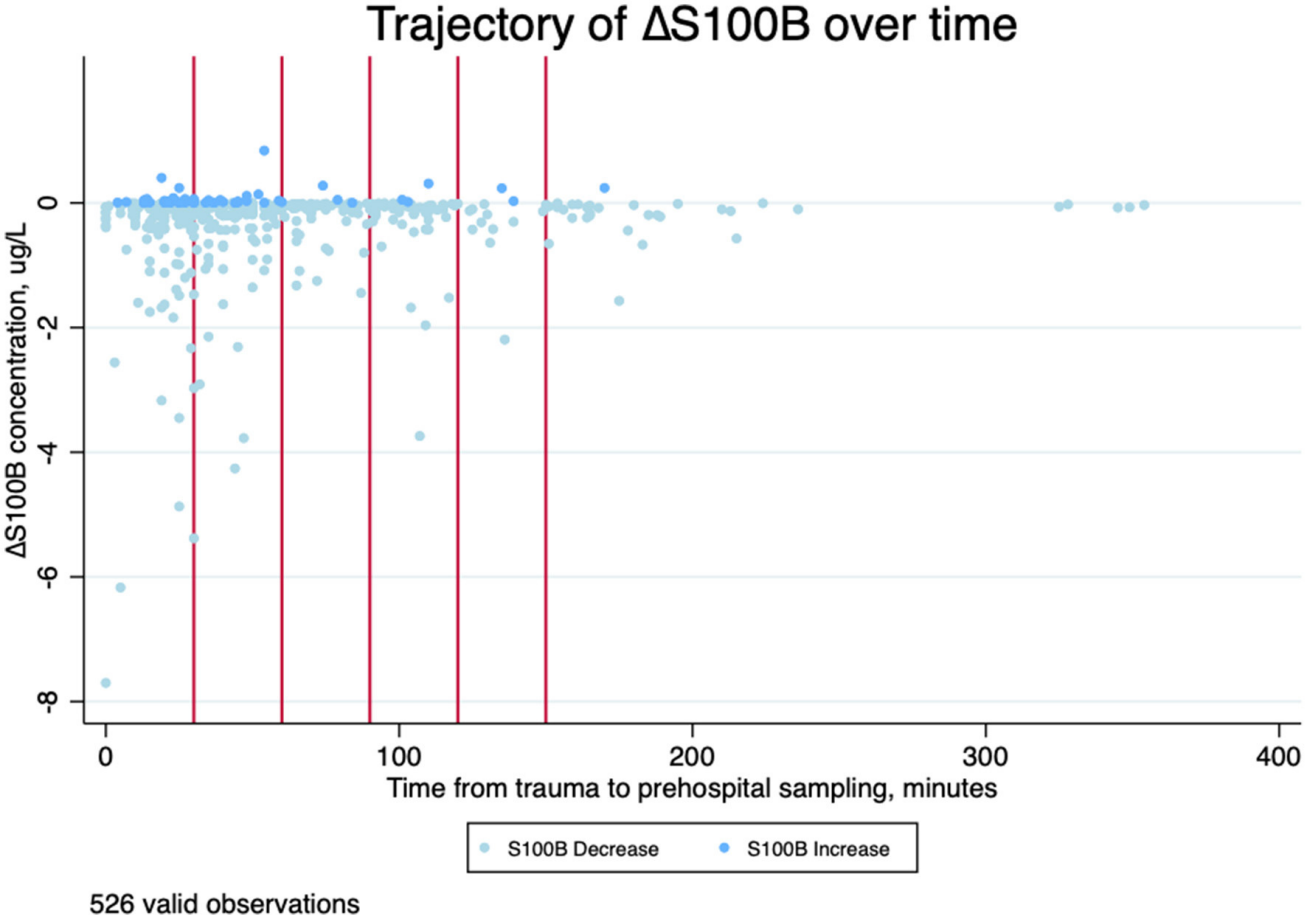
Scatterplot of time from trauma to blood sampling in minutes (the first 6 h) and change in serum S100B concentrations in μg/l (S100B) from prehospital to in-hospital sampling stratified by trajectory (decreasing or increasing value). The red lines indicate 30, 60, 90, 120, and 150 min after trauma. *N* = 526.

S100B trajectory between prehospital and in-hospital concentrations (S100B) are presented in Table 3. Concentrations decreased from prehospital to in-hospital samples in 534/592 (90.2%) of patients with TBI with a mean decrease of S100B concentration was−0.34 μg/L (95%CI −0.41; −0.28). Increasing concentrations were mainly seen in patients with early sampling within the first hour after trauma. The graphical representation of the S100B trajectory associated with time from trauma to first sampling (prehospital) is displayed in Figure 4.

**Table 3:** Trajectory of S100B between prehospital and in-hospital concentrations (S100B increase or decrease) stratified by (1) initial GCS, (2) intracranial lesion, and (3) time from trauma to first (prehospital) sampling in 30 min intervals, *N* = 592.

**Variable**	**S100B increase**	**S100B decrease**
Total observations, n/N (%)	58/592 (9.8%)	534/592 (90.2%)
Change in S100B concentration, mean μg/L (95%CI)	0.13 (0.01;0.25)	−0.34 (−0.41; −0.28)
Percentwise change in concentration, mean % (95%CI)	38.1 (16.5;59.7)	−41.5 (−43.3: −39.7)
- first sample< 60 min from trauma	33.9 (7.0;60.8)	−41.1 (−43.4; −38.7)
- first sample > 60 min from trauma	51.4 (28.3;74.6)	−42.3 (-45.2; −39.3)
*GCS, n/N (%)*		
Initial GCS 14–15 (mild)	57/566 (10.1)	509/566 (89.9)
Initial GCS 9–13 (moderate)	1/20 (5.0)	19/20 (95.0)
Initial GCS 3–8 (severe)	0/6 (0)	6/6 (100.0)
*Intracranial lesion, n/N (%)*		
Yes	3/39 (7.6)	36/39 (92.3)
*Time from trauma to first sample, N =528*		
0–30 min	29/193 (15.0)	164/193 (85.0)
30–60 min	17/161 (10.6)	144/161 (89.4)
60–90 min	3/70 (4.3)	67/70 (95.7)
90–120 min	3/57 (5.3)	54/57 (94.7)
120–150 min	3/47 (6.4)	44/47 (93.6)

*GCS, Glasgow Coma Scale; CI, Confidence interval*.

Changes in S100B concentrations from prehospital to in-hospital sampling crossed the guideline cut-off at 0.10 μg/L in 90/592 (15.2%) of decreasing trajectories and in 2/592 (0.3%) of increasing trajectories. No S100B concentration trajectories of patients suffering intracranial lesions or death crossed the guideline cut-off, but remained elevated at both prehospital and in-hospital sampling.

**Figure 5:**
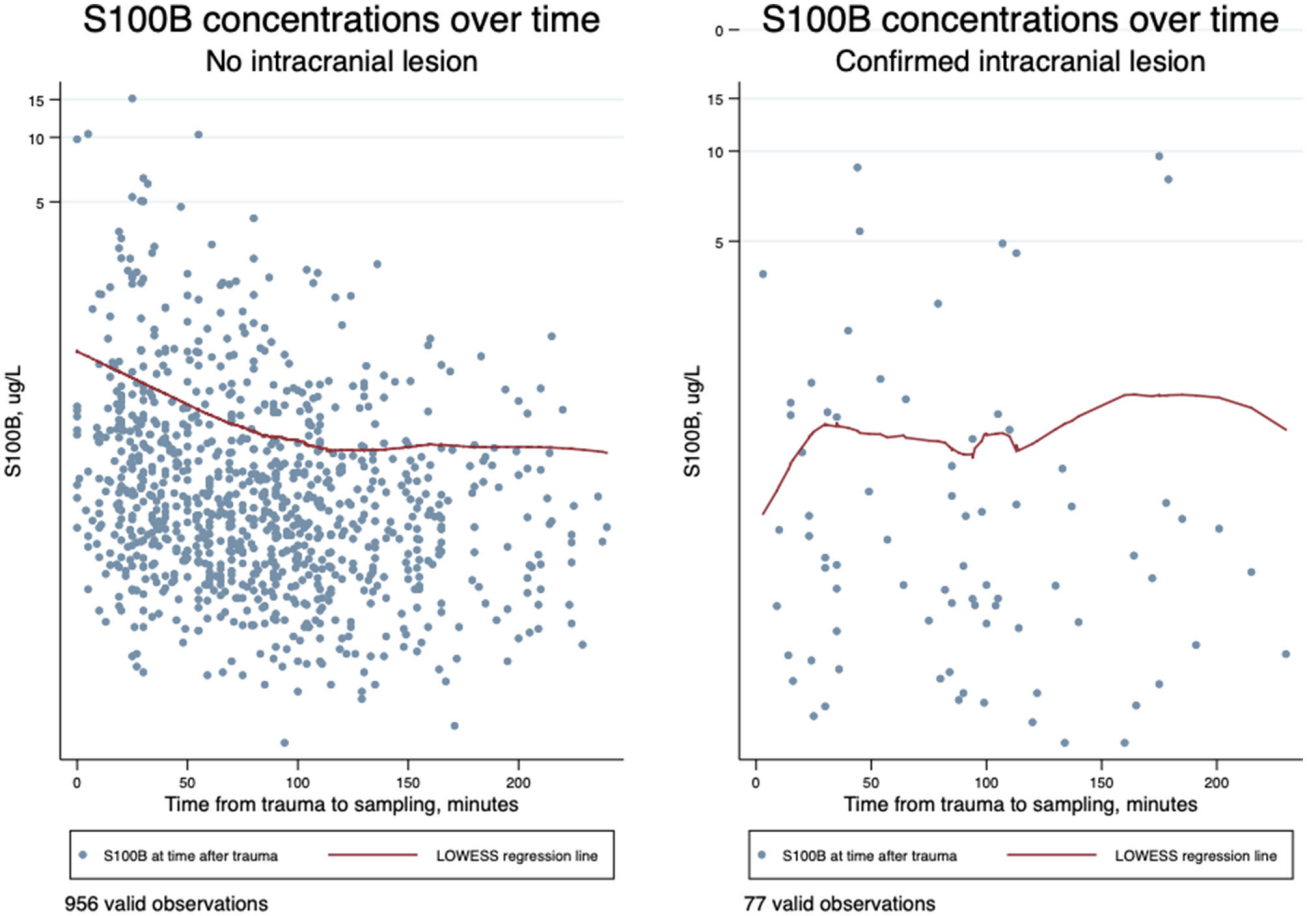
Scatterplot of time from trauma to blood sampling in minutes (the first 4 h) on an ordinary scale and serum S100B concentrations in μg/l on a logarithmic scale overlaid with the locally weighted scatterplot smoothing (LOWESS) regression line demonstrating temporal changes of S100B values in samples from patients without intracranial lesions (N = 956) and in samples from patients with a confirmed intracranial lesion (*N* = 77).

The temporal changes of all serum S100B concentrations over time overlaid with the locally weighted scatterplot smoothing (LOWESS) regression line are presented in Figure 5. The figure illustrates S100B concentrations to decline early after the trauma but with a slight incline before the decline in patients with confirmed intracranial lesions.

## Discussion

This *post hoc* descriptive study evaluated the temporal changes of serum S100B in two consecutive samples from patients with head trauma sampled during the initial phase after trauma in the prehospital setting and again upon in-hospital admission.

In 90% of the patients, S100B concentrations decreased from prehospital sampling to in-hospital sampling with a mean decrease of −0.34 μg/L. Patients with increasing trajectories were generally sampled very early after trauma and no increases occurred after 180 min. Patients suffering from intracranial lesions presented with both increasing and decreasing trajectories.

Current knowledge on early dynamics and temporal changes of S100B was reviewed by Thelin et al. (12). In their systematic review, a decline in S100B was found in 30 studies whereas 5 studies described a slight increase before the decrease. These findings are in concordance with the findings of this current study, but none of the previous studies were based on a broad material with samples taken at the earliest time possible—i.e., in the ambulance within the first hour after trauma. The few previous studies that came the closest to such ultra-early sampling mainly consist of older studies with a low number of observations or case reports (18–20), and a single newer study by Welch et al. (21). Welch and colleagues reported results of repeated sampling of head trauma patients (90% cases with GCS 14-15) the first 24 h after trauma, with 47% of samples drawn within 6 h—but no samples collected already in the prehospital phase. Welch et al. report S100B to decline early; already within the first hour in patients with negative CT examinations and after 3 hours following a slight incline in patients with positive CT examinations. They report a minor mean S100B decrease of 0.12 μg/L corresponding to the longer time from trauma to sampling. To our knowledge, Welch et al. used the same S100B assay (Roche, Cobas6000 chemiluminescence immunoassay using a Cobas^®^ e602 analyzer) as we did in this current study. The results must be considered comparable. However, Enochson et al. used a monoclonal two-site immunoradiometric assay (Sangtec 100; AB Sangtec Medical, Bromma, Sweden), and Vos et al. used 2-site luminometric immunoassays with 1 incubation step performed on the STAT IntraOperative platform (Future Diagnostics, Wijchen, Netherlands). This means that the analytical methods are not directly comparable, the different methods could have different cut-off values and the diagnostic accuracy may vary.

In this current study, we used the hemolysis index as a measure of sample quality. The HIL levels proved all samples to contain concentrations below the clinically accepted value for hemolysis in S100B analysis. We observed a rather small difference in hemolysis index between prehospital and in-hospital samples, why we do not consider the effect of HIL in the analysis.

In patients with increasing S100B concentrations in the present study, the mean increase was lower than the decrease (0.13 μg/L vs. −0.34 μg/L) and the majority of increasing samples (83.6%) were collected within 60 min of trauma. The mean percentage deviation between prehospital and in-hospital samples was all well above the maximum monthly coefficient of variance of the used analyzer at 7.1% for both increasing and decreasing trajectories, and the variation between consecutive samples cannot solely be ascribed uncertainty of measurements.

Only (3/39) 8% of the patients suffering from intracranial lesions had increasing values. The LOWESS regression line in Figure 5 however suggests a possible incline, before the decline as a tendency in these patients. Even though Welch and colleagues sampled the study subjects later after trauma, they report a slight increase in S100B early after trauma in patients with findings on CT examination as well, whereas no increase was observed in patients without findings (21).

The 2014 Scandinavian Neurotrauma Committee guidelines for the initial management of adult patients with minimal to moderate head injury recommend against the use of S100B for the rule-out of intracranial lesions in patients in anticoagulant treatment, patients aged >65 years in antiplatelet treatment, and in patients with extracranial injuries (large fractures of tubular bones) (22). However, when plotting the patterns of S100B concentrations against time from trauma to sampling for patients above the age of 65 years, in antiplatelet/-coagulation treatment, and/or with extracranial tubular bone fractures in addition to head trauma and even alcohol-intoxicated, we observed no difference in the patterns. Also, we observed no patients with S100B <0.10 μg/L suffering intracranial lesion when allowing these subgroups into the analysis. Thus, the biomarker S100B may be more versatile for rule-out than expected. The decision to recommend against the use of S100B for the rule-out of intracranial lesions in patients in anticoagulant treatment, patients aged >65 years in antiplatelet treatment, and in patients with extracranial injuries in the Scandinavian guideline was consensus-based. Further investigation on the subgroups may provide the basis for evidence-based guidelines on the subject.

In a previously published work, we reported the diagnostic accuracy of prehospital serum S100B to rule out intracranial lesions in patients suffering mild TBI (initial GCS 14-15) (13). We demonstrated that S100B was safe for prehospital rule-out of intracranial lesions, but with lower specificity (15%) in prehospital samples than in-hospital samples (31%). The cost-effectiveness of a prehospital rule-out strategy was not evaluated and it might be speculated if other novel biomarkers, such as glial fibrillary acidic protein (GFAP) and ubiquitin carboxy-terminal hydrolase L1 (UCH-Ll), may outperform S100B in the ultra-early diagnostics of patients with TBI. These biomarkers are primarily investigated with later, in-hospital sampling, and future research should investigate if GFAP and UCH-L1 rise and reach peak values in a timely manner for prehospital use (12, 21). In practice, prehospital biomarker sampling would be useful when certain clinical information (e.g., CT examination) is not available at the time of assessment. For a more widespread application, whole blood assays for point-of-care analysis would be beneficial.

### Strengths and Limitations

A strength to the PreTBI studies was the clinical, single-blinded, prospective, observational, multicenter design with prehospital inclusion of unselected patients with TBI in the entire Prehospital Emergency Medical Services, Central Denmark Region. The setup strengthens the external validity of the findings as it reflects the every-day work of ambulance personnel. Also, the risk of detection bias was reduced as the outcome assessors were blinded to biomarker values (15–17).

A limitation to the study is the low number of patients with moderate and severe TBI included. Fewer patients than expected were eligible for inclusion and in addition, obtaining *post hoc* informed consent proved more difficult than expected. Further, not all of the included patients with severe TBI had a CT scan performed. Also, we only recorded data on isolated vs. multiple trauma, and not exact data on blunt vs. penetrating trauma. Another limitation to the study is the high exclusion rate mainly driven by insufficient blood sampling. Insufficient blood sampling covers lack of timely handover of the prehospital sample, missed in-hospital blood sampling, and/or invalid marking of samples with patient identification and timestamps. A higher percentage of the excluded patients had initial GCS <13. Our results are more applicable to mild TBI patients than moderate and severe TBI patients as this population is under-represented in our sample due to selection bias.

Even though this study was not designed to determine complex kinetic modeling, this *post hoc* description of the temporal changes of S100B providing T_0_ as the time of trauma, is a pragmatic attempt to address the practical and ethical challenges in kinetic studies of human serum biomarkers. The variations in sampling timepoints due to these challenges complicate further kinetic modeling, but are on the other hand reflective of how biomarkers are sampled in real-life clinical practice.

## Conclusion

S100B concentrations seem to decline already within the first hours of head trauma and potentially peak earlier than expected from previous studies of temporal changes in human TBI. Further studies are needed to investigate the ultra-early temporal changes in the subgroups of patients aged > 65 years, and/or in antiplatelet/-coagulant treatment.

## Data Availability Statement

The datasets presented in this article are not readily available because the protection of personal data requires reasonable request and relevant permissions from Danish authorities according to Danish law prior to distribution of data. Requests to access the datasets should be directed to S-CS, soseid@rm.dk.

## Ethics Statement

The studies involving human participants were reviewed and approved by the Regional Scientific Ethical Committee System, Central Denmark Region. The patients/participants provided their written informed consent to participate in this study.

## Author Contributions

S-CS, MT, NJ, HK, and A-MB designed the study. S-CS and JK were responsible for data collection and administration of the biological bank. MF was responsible for all biochemical analyses. MT and NJ conducted the endpoint committee review of all medical patient records. S-CS and MT conducted the analysis and interpretation of data. S-CS drafted the manuscript. JK, NJ, HK, MF, A-MB, and MT revised it critically for important content. All authors approved the final version of the manuscript prior to publication.

## Funding

The PreTBI I, II, and III studies were entirely initiated by the research group. No commercial companies were involved in the design or initiation of the study. The salary for primary investigator amounts to the regular PhD salary throughout the study period. The project was financially supported by external foundations: The A.P. Møller and Chastine Mc-Kinney Møller Foundation for General Purposes (Medical Foundation) (DKK 60.000), Holger and Ruth Hesse Memorial Foundation (DKK 64.000), Central Denmark Region Health Research Foundation (DKK 480.000), The Civil Affairs Agency, Danish Ministry of Justice (Offerfonden) (DKK 600.000), and The Danish Air Ambulance (480.000). The Regional Scientific Ethical Committee System was notified of all funding during the study period.

## Conflict of Interest

The authors declare that the research was conducted in the absence of any commercial or financial relationships that could be construed as a potential conflict of interest.

## Publisher's Note

All claims expressed in this article are solely those of the authors and do not necessarily represent those of their affiliated organizations, or those of the publisher, the editors and the reviewers. Any product that may be evaluated in this article, or claim that may be made by its manufacturer, is not guaranteed or endorsed by the publisher.
