# Supplementary material for: Temporal Changes in Serum S100B Levels From Prehospital to Early In-Hospital Sampling in Patients Suffering Traumatic Brain Injury
Source: Front Neurol. 2022 Apr 8;13:800015. doi: 10.3389/fneur.2022.800015 (PMC9033508; doi:10.3389/fneur.2022.800015)
Supplement: Supplementary file 1 [file Data_Sheet_1.docx]

**Supplemental Material 1**

**Figure S1**

Scatterplot of time from trauma to blood sampling in minutes on ordinary scale (the first 4 hours) and serum S100B concentrations in μg/L on a logarithmic scale stratified by initial GCS 14-15, GCS 9-13 and GCS 3-8. The red line indicating guideline cut-off at 0.10 μg/L. N = 508 (valid timestamps the first 4 hours).

**
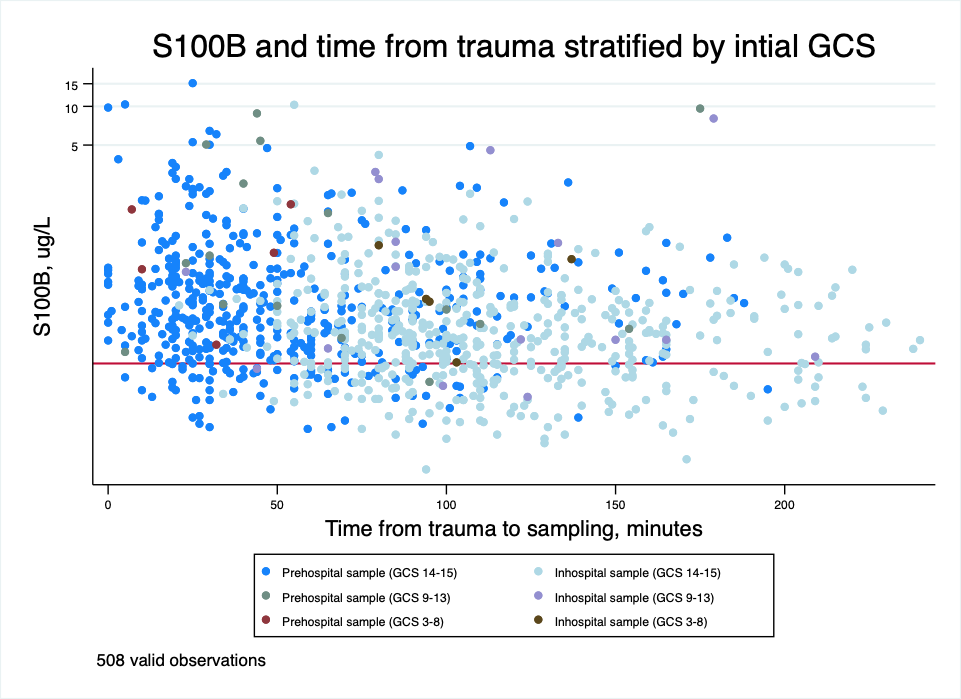
**
